# Nutritional Knowledge, Dietary Habits, and Nutritional Status of Patients with Chronic Kidney Disease According to Disease Stage

**DOI:** 10.3390/nu18071109

**Published:** 2026-03-30

**Authors:** Filip Siódmiak, Sylwia Małgorzewicz

**Affiliations:** 1 Student Circle Clinical Nutrition and Dietetics, Department of Clinical Nutrition, Medical University of Gdańsk, Skłodowskiej-Curie 3a Street, 80-211 Gdansk, Poland; filip777@gumed.edu.pl; 2 Department of Clinical Nutrition, Medical University of Gdańsk, Skłodowskiej-Curie 3a Street, 80-211 Gdansk, Poland

**Keywords:** chronic kidney disease, nutritional education, diet therapy, nutritional status, CKD

## Abstract

**Background/Objectives**: Appropriate nutritional management constitutes one of the key elements of conservative treatment and renal replacement therapy in patients with chronic kidney disease (CKD). The level of patients’ nutritional knowledge may significantly influence adherence to dietary recommendations, the rate of disease progression, and the frequency of complications. The aim of this study was to assess the level of nutritional knowledge, dietary habits, adherence to dietary recommendations, and nutritional status of patients with CKD according to disease stage. **Methods**: This cross-sectional study was conducted among 98 adult patients diagnosed with CKD. A questionnaire assessing nutritional knowledge and dietary behaviors was administered. An overall nutritional knowledge score was calculated based on eight questionnaire items assessing nutritional knowledge. Nutritional status was evaluated using the Subjective Global Assessment (SGA) and the Simplified Nutritional Appetite Questionnaire (SNAQ). Anthropometric, clinical, and biochemical data were collected. Statistical analysis was performed using tests appropriate to the data distribution. **Results**: The level of nutritional knowledge varied and was dependent on CKD stage. Patients in more advanced stages of the disease demonstrated significantly higher awareness of dietary recommendations compared with those in earlier stages. The median nutritional knowledge score was 6 points, with 46.9% of participants demonstrating insufficient knowledge (<6 points) and 53.1% achieving adequate knowledge (≥6 points). The greatest knowledge deficits concerned the control of phosphorus, potassium, sodium, and fluid intake. Discrepancies were also observed between declared knowledge and actual dietary behaviors. Good nutritional status (SGA A) was identified in 73 patients, risk of malnutrition or moderate malnutrition (SGA B) in 22 individuals, and severe malnutrition (SGA C) in 3 patients. SNAQ indicated good appetite in the study population, with an average consumption of three meals per day, and identified a risk of weight loss in 6% of patients. Overweight and obesity were present in more than half of the study population, while underweight was observed in 4%. **Conclusions**: Nutritional knowledge among patients with CKD remains insufficient, particularly in the early stages of the disease. The findings highlight the necessity of early and systematic implementation of individualized nutritional education as an integral component of slowing disease progression.

## 1. Introduction

Chronic kidney disease (CKD), according to the 2012 definition of Kidney Disease: Improving Global Outcomes (KDIGO), is defined as abnormalities of kidney structure or function, present for at least three months, with health implications [1]. CKD represents an increasing public health problem. Globally, it is estimated to affect 8–16% of the general population, corresponding to nearly 850 million individuals, and contributes to approximately 2.4 million deaths annually. In Poland, CKD affects approximately 4.7 million individuals, meaning that the disease is diagnosed in roughly one in eight adults [1].

Progressive deterioration of kidney function leads not only to disturbances in water-electrolyte and acid–base balance but also to intensification of catabolic processes [2]. Inadequately treated and monitored CKD promotes the development of protein–energy wasting (PEW), accelerated disease progression, increased hospitalization rates, and elevated mortality risk [3,4,5].

Comprehensive care for patients diagnosed with CKD is not limited to regular nephrology follow-up. A crucial component of therapeutic management affecting both disease progression and quality of life is systematic dietary care provided by a qualified dietitian and tailored to the individual needs of the patient [2,6,7].

According to current guidelines from the Kidney Disease Outcomes Quality Initiative (KDOQI) and European Society for Clinical Nutrition and Metabolism (ESPEN), nutritional recommendations for patients with CKD should be adjusted to the stage of disease, treatment modality, and individual clinical condition [2,3,4,5,6,7]. Despite this individualization, several universal principles of nutritional management can be identified:

- Ensuring adequate energy intake, usually 30–35 kcal/kg body weight per day, to prevent catabolism and muscle mass loss [2,6];
- Controlling protein intake, adjusted to ideal body weight and disease stage [2,6,8];
- Restricting sodium intake, typically to approximately 2 g per day (equivalent to 5 g of salt), to improve blood pressure control and fluid status [2,9];
- Monitoring electrolyte intake according to laboratory results to prevent cardiac arrhythmias [9,10];
- Preventing deficiencies of vitamins and minerals, including vitamin D, iron, zinc and B vitamins [6,7];
- Optimizing hydration according to CKD stage and urine output [2,11].

Nutritional intervention in patients with CKD, when appropriately implemented, may contribute not only to slowing disease progression but also to improving quality of life, reducing metabolic complications, and decreasing cardiovascular risk [12,13].

During conservative treatment, encompassing CKD stages 3–5, the primary goal of individualized diet therapy is to slow disease progression and limit metabolic complications [2]. In patients with stages 1–2, nutrient intake, including protein, should be consistent with recommendations for the general population (approximately 0.8 g/kg body weight per day). According to KDOQI guidelines, in individuals with a persistent glomerular filtration rate (GFR) below 60 mL/min/1.73 m^2^ (from stage 3 CKD onward), who are well-nourished and without metabolic disturbances, recommended protein intake is approximately 0.6 g/kg body weight per day, provided that adequate energy intake is ensured [2,6]. In selected clinical cases, a very-low-protein diet (VLPD; 0.3–0.4 g/kg body weight per day) supplemented with ketoanalogues of amino acids may be considered [2]. In situations where patients with CKD present with significant inflammation or features of malnutrition, current ESPEN recommendations allow increasing protein intake up to 1.3 g/kg body weight per day [6,8].

Mineral intake (potassium, phosphorus) should be determined individually based on current serum concentrations. During conservative treatment, a relatively high daily fluid intake of approximately 2–3 L from all sources is recommended, unless contraindications such as heart failure, fluid overload, oliguria, or anuria are present. Increased fluid intake at this stage may be associated with reduced uremic toxin concentration, slower CKD progression, and improved quality of life [2,11].

Initiation of renal replacement therapy, including hemodialysis, peritoneal dialysis, or kidney transplantation, requires modification of dietary recommendations. In dialysis patients, increased protein intake is recommended due to amino acid losses in dialysate and enhanced catabolic processes related to blood contact with the dialysis membrane [2,7,8]. During this period, recommended protein intake is 1.0–1.2 g/kg body weight per day, and in the presence of inflammation or malnutrition, it may be increased to 1.4 g/kg body weight per day [2,7,8]. Patients undergoing renal replacement therapy are particularly susceptible to fluctuations in fluid status. Recommended daily fluid intake depends on residual diuresis, clinical condition, and interdialytic weight gain, which should not exceed 2–3 kg (approximately 3–5% of body weight) [7,11]. Exceeding these values is associated with an increased risk of fluid overload and cardiovascular complications.

A key objective in water-electrolyte management is maintaining euvolemia. Both fluid overload, leading to left ventricular hypertrophy and hypertension, and dehydration, resulting in deterioration of residual filtration and hypotension, are associated with poorer prognosis and increased mortality [9,11]. Optimization of fluid balance requires close cooperation between the nephrology and dietetic teams, as well as regular patient education regarding fluid intake control [7].

Properly conducted nutritional education and regular assessment of nutritional status constitute essential elements of comprehensive CKD care. The goal of education is to provide reliable knowledge and develop practical skills necessary for informed dietary decision-making that supports slowing disease progression and reducing complication risk. Regular nutritional assessment enables early identification of malnutrition risk or established malnutrition and the timely implementation of appropriate dietary modifications. In the long term, such education contributes to improved nutritional status, enhanced quality of life, and greater patient control over the course of the disease [7,11].

Despite the established importance of diet in CKD management, patients’ nutritional knowledge and adherence to dietary recommendations remain insufficient, particularly in the early stages of the disease. Therefore, assessing nutritional knowledge, dietary habits, and nutritional status across different CKD stages is essential to improve clinical outcomes and guide effective dietary education strategies.

## 2. Materials and Methods

This cross-sectional study included 98 adult patients with chronic kidney disease who were admitted to the Nephrology Department for routine clinical examinations. The study protocol was approved by the Bioethical Committee (approval no. KB/343-183/2025).

The study group consisted of 55 women and 43 men. The mean age of the patients was 54 ± 17 years. Each participant was informed about the purpose and course of the study and was given the opportunity to ask questions before providing written informed consent. Inclusion criteria were: age ≥ 18 years, diagnosis of CKD, and voluntary written consent to participate in the study. The most frequently reported comorbidities were arterial hypertension (*n* = 56; ~65%) and diabetes mellitus (*n* = 10; ~18%). Cardiovascular diseases (including myocardial infarction, atrial fibrillation, atherosclerosis, and cardiomyopathy) were observed in approximately 10% of patients. Less frequent conditions included tuberous sclerosis (*n* = 7), polycystic kidney disease (*n* = 3), glomerulonephritis (*n* = 3), gout (*n* = 3; ~5%), autoimmune diseases such as systemic lupus erythematosus (~6%), thyroid disorders (~6%), and anemia (~5%). Single cases included nephrotic syndrome and other less common conditions.

### 2.1. Nutritional Status

Nutritional status was assessed using two tools: the Subjective Global Assessment (SGA) and the Simplified Nutritional Appetite Questionnaire (SNAQ).

SGA includes the evaluation of body weight changes over the previous 2 weeks and 6 months, changes in the quantity and form of meals consumed, the presence of gastrointestinal symptoms, level of physical performance, the impact of disease on energy requirements, and elements of physical examination. Based on the overall assessment, patients were classified into one of three categories:

A—well nourished;

B—suspected malnutrition or moderate malnutrition;

C—severe malnutrition.

Additionally, the Simplified Nutritional Appetite Questionnaire (SNAQ) was used. It consists of four questions regarding appetite, feeling of fullness after meals, usual number of meals per day, and changes in taste perception. Each question is rated on a five-point scale (1–5), yielding a total score ranging from 4 to 20 points. Lower scores indicate a greater risk of weight loss; a score ≤14 points is considered an indicator of increased risk of losing at least 5% of body weight within the next 6 months. In the study group, this criterion was met by 5 patients.

### 2.2. Anthropometric Measurements

Anthropometric measurements were performed in all participants and included body weight, height, calf circumference (CC), and calculation of body mass index (BMI, kg/m^2^). A calf circumference was measured using a measuring tape in the dominant limb, with the patients in a seated position and a 90° angle maintained between the calf and thigh. The measurement was taken at the widest point of the calf, with slight adjustments (±1 cm) to determine the maximum circumference. A cut-off value of ≥31 cm was adopted as an indicator of abnormal nutritional status [14].

### 2.3. Laboratory Parameters

As part of routine examinations performed during planned hospitalization, the following laboratory tests were measured in the hospital’s central laboratory: creatinine, urea, potassium, sodium, phosphorus, and calcium.

The estimated glomerular filtration rate (eGFR) was calculated using the MDRD formula and served as the basis for the classification of patients into specific CKD stages.

### 2.4. Nutritional Knowledge and Habits

Nutritional knowledge and dietary behaviors were assessed using a structured questionnaire based on a previously applied instrument developed for a nationwide study conducted in a population of patients with chronic kidney disease in Poland and published in Forum Nefrologiczne [15].

In the present study, the questionnaire was adapted to specifically address dietary aspects and eating habits in patients with CKD. Additionally, supplementary questions regarding the use of oral nutritional supplements (ONSs) and dietary supplements were included.

The final version of the questionnaire consisted of 15 items, including 8 assessing nutritional knowledge, 4 related to dietary habits, and 3 evaluating adherence to dietary recommendations.

To assess overall nutritional knowledge, a composite knowledge score was calculated based on eight questionnaire items. The items included in the score covered key aspects of dietary management in CKD, including protein and energy requirements, fluid intake, and the dietary content of potassium, calcium, phosphorus, and sodium. Additionally, the questionnaire assessed patients’ awareness of culinary techniques used to reduce potassium and phosphorus content in foods.

Each correct answer was assigned 1 point, resulting in a total score ranging from 0 to 8 points. Based on the median value, nutritional knowledge was categorized as insufficient (<6 points) or adequate (≥6 points).

The internal consistency of the questionnaire was assessed using Cronbach’s alpha coefficient, yielding a value of α = 0.53. This indicates moderate internal consistency, which may be explained by the multidimensional nature of the questionnaire and the inclusion of items addressing different domains (nutritional knowledge, dietary habits, and supplement use). Therefore, the questionnaire should be considered an exploratory tool.

#### Statistical Analysis

Statistical analysis was performed using Statistica 13.3 PL. Continuous variables were presented as mean ± standard deviation (SD) or median (interquartile range), depending on the distribution of data, while categorical variables were expressed as numbers and percentages. The normality of distribution was assessed using the Shapiro–Wilk test. Differences between groups according to CKD stage were analyzed using one-way analysis of variance (ANOVA) for normally distributed variables or the Kruskal–Wallis test for non-normally distributed variables. For categorical variables, the chi-square test was applied.

Post hoc analyses were performed using Tukey’s test or Dunn’s test, as appropriate. To identify factors associated with nutritional knowledge, multivariable linear regression analysis was performed. The selection of covariates was based on clinical relevance and statistical considerations. A *p*-value < 0.05 was considered statistically significant.

## 3. Results

### 3.1. Assessment of Nutritional Status

**Figure 1:**
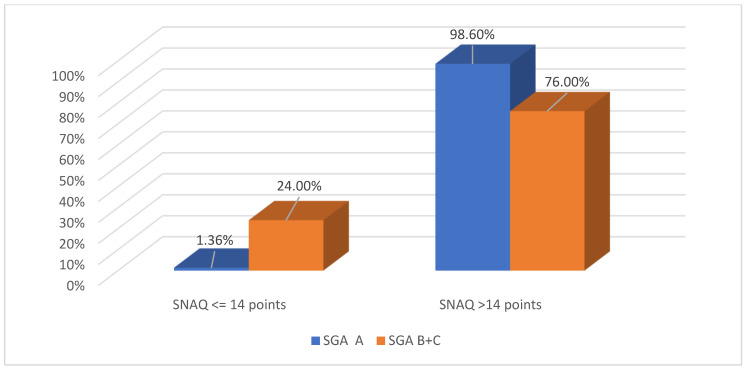
Results of SNAQ in well-nourished and malnourished CKD patients.

Assessment based on SGA and SNAQ demonstrated varying degrees of malnutrition risk in the study population, depending on CKD stage and treatment modality. In the study population, normal nutritional status (SGA = A) was identified in 73 patients (74%), risk of malnutrition or moderate malnutrition (SGA = B) in 22 patients (23%), and severe malnutrition (SGA = C) in 3 patients (4%). SNAQ scores ≤14 were more frequently observed in patients with impaired nutritional status (Figure 1).

SNAQ scores ranged from 5 to 20 points (mean: 17). The largest proportion of patients achieved high scores:

- 20 points–21 patients (22%);
- 19 points–20 patients (21%);
- 18 points–19 patients (20%).

Low scores (≤14 points), indicating risk of weight loss and potential malnutrition, were recorded in only 7 individuals.

The most frequently selected response to the SNAQ question regarding appetite (“My appetite is”) was “very good,” reported by 42% (*n* = 41) of patients. “Good” was selected by 30% (*n* = 29). Only 7% (*n* = 6) rated their appetite as “poor” or “very poor”.

Regarding the feeling of fullness during meals, 68% (*n* = 67) responded, “I rarely feel full after eating a meal.” Only 4% of participants (*n* = 4) reported “I feel full after eating one-third of a meal” or “I feel full after eating only a few bites”.

Fifty-three percent (*n* = 52) answered “very good” to the question “Food tastes good to me.” Eight percent (*n* = 8) rated it as “moderately,” and 3% (*n* = 3) as “very bad”.

Sixty-two percent of participants consumed more than three meals per day, whereas only 7% (*n* = 7) reported consuming two meals per day.

### 3.2. Anthropometric Measurements

Anthropometric measurements according to CKD stage are presented in Table 1.

**Figure 2:**
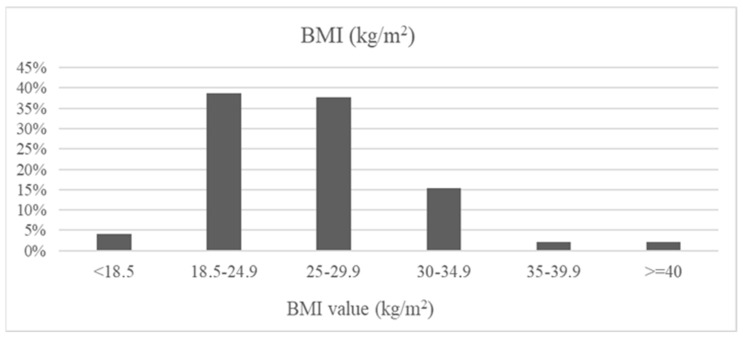
BMI values in the study population.

BMI values ranged from 17.3 to 42.7 kg/m^2^. Underweight was observed in 4% of participants (*n* = 4), normal body weight was observed in 39% (*n* = 38), and overweight was observed in 38% (*n* = 37). Class I obesity was present in 15% (*n* = 15), whereas class II obesity and morbid obesity were each diagnosed in 2% of patients (*n* = 2 per category). These findings indicate that excess body weight and obesity affected more than half of the study population (Figure 2).

### 3.3. Calf Circumference

Mean calf circumference values ranged from 28 to 47 cm, with a mean of 35.9 cm. Normal calf circumference (≥31 cm) was observed in 94 patients (96%). The highest mean calf circumference was observed in patients with CKD stage 1.

No statistically significant differences were found in body weight, height, body mass index (BMI), or calf circumference between CKD stages (ANOVA, *p* > 0.05 for all comparisons).

### 3.4. Biochemical Parameters

Analysis of biochemical parameters across CKD stages demonstrated statistically significant differences in glomerular filtration rate (GFR), serum creatinine concentration, and serum levels of phosphorus, potassium, and calcium. Disease-characteristic decrease in GFR and increase in serum creatinine were observed. Concurrently, progressive disturbances in mineral metabolism were noted, particularly in relation to phosphorus, calcium, and potassium levels (Table 2). Among patients with CKD stage 5D (*n* = 11), compared with those with CKD stage 5 treated conservatively (*n* = 22), higher potassium concentrations were observed (5.44 ± 1.03 vs. 4.75 ± 0.43 mmol/L). Analysis of biochemical parameters across CKD stages demonstrated statistically significant differences in glomerular filtration rate (GFR), serum creatinine concentration, and serum levels of phosphorus, potassium, and calcium (Kruskal–Wallis test, *p* < 0.05).

### 3.5. Analysis of the Nutritional Questionnaire

#### 3.5.1. Nutritional Knowledge

As many as 90% of respondents (*n* = 88) considered diet an important component of CKD treatment. Ten percent believed that diet did not play a key role in disease management.

The majority of participants (73%, *n* = 72) were aware of the importance of appropriate protein intake in CKD. Only 28% (*n* = 27) knew their current energy requirements, whereas 72% (*n* = 71) did not possess such knowledge.

**Figure 3:**
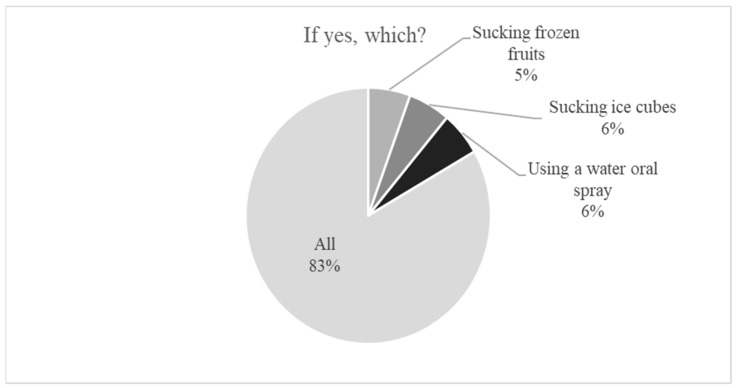
Level of knowledge regarding thirst-reduction techniques among CKD patients.

A substantial majority (81%, *n* = 79) did not know methods to reduce thirst. Knowledge of at least one method was declared by 19%, including 5 conservatively treated patients and 14 patients undergoing or having previously undergone renal replacement therapy. Among these, 83% (*n* = 16) were able to list all major methods to reduce thirst, whereas 21% (*n* = 4) provided only one method (Figure 3).

The median nutritional knowledge score in the study population was 6 points. A total of 46 participants (46.9%) scored below 6 points, while 52 (53.1%) achieved scores of 6 points or higher. The knowledge score differed significantly across CKD stages (χ^2^ = 38.59, df = 24, *p* = 0.03). A significant association was also observed between knowledge score and age (χ^2^ = 386.5, df = 318, *p* = 0.005).

#### 3.5.2. Oral Nutritional Supplements

Oral nutritional supplements classified as food for special medical purposes (FSMP) were used by 22% of participants (*n* = 21). The remaining 78% (*n* = 77) had never used such preparations. The most frequently reported products were high-protein FSMPs or products dedicated to patients with impaired kidney function.

#### 3.5.3. Knowledge of Sources of Nutrients

Analysis of knowledge regarding sources of sodium, potassium, phosphorus, and calcium revealed substantial variability. Thirteen percent of participants were unable to identify any correct source of these minerals.

Dairy products (*n* = 69) were most commonly identified as sources of calcium. Vegetables and fruits (*n* = 59), including tomatoes (*n* = 9), were most frequently mentioned as potassium-rich foods. The most frequently cited sources of sodium were processed meats (*n* = 38), canned foods (*n* = 26), salty snacks (*n* = 22), and yellow cheese (*n* = 14). Fish (*n* = 23), meat (*n* = 18), eggs (*n* = 8), and cola beverages (*n* = 8) were most commonly identified as sources of phosphorus.

**Figure 4:**
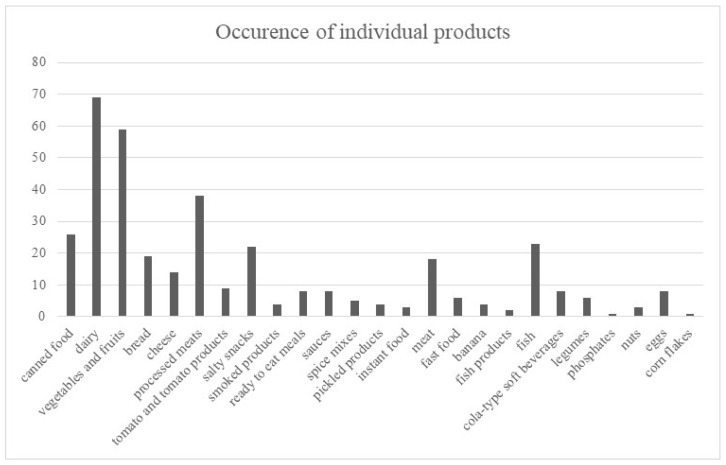
Sources of sodium, potassium, calcium, and phosphorus.

None of the patients were able to simultaneously identify products that are sources of both sodium and phosphorus, such as processed meat or fish products and canned foods. Other reported products are presented in Figure 4.

#### 3.5.4. Dietary Supplements

**Figure 5:**
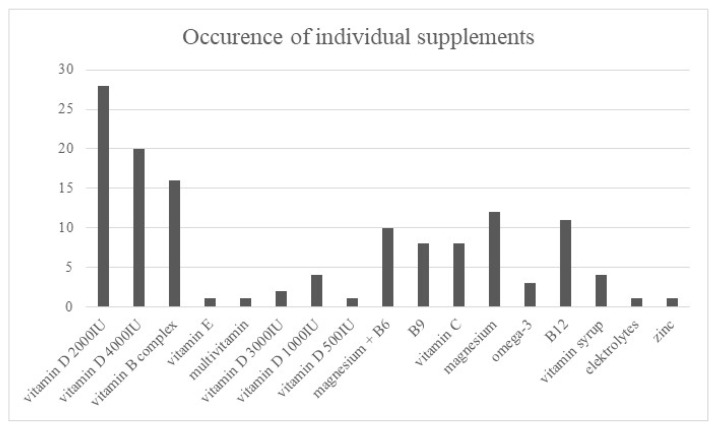
Dietary supplements used by participants.

Thirty-two percent of participants (*n* = 31) were not currently using any dietary supplements. The most commonly reported supplements were vitamin D at doses of 2000 IU (*n* = 28) and 4000 IU (*n* = 20), B-complex vitamins (*n* = 16), vitamin C at doses ranging from 200 to 2000 mg/day (*n* = 12), magnesium preparations (*n* = 11), folic acid (*n* = 10), and omega-3 fatty acids at 1 g/day (*n* = 8). Other supplements are shown in Figure 5.

#### 3.5.5. Dietary Habits

Thirty-five percent of patients (*n* = 34) reported salting food at a level similar to the period before diagnosis. Reduction or complete elimination of sodium chloride was declared by 65% (*n* = 64).

Regarding cereal products, 44% (*n* = 43) preferred whole-grain products, whereas 56% (*n* = 55) chose refined-grain products.

**Figure 6:**
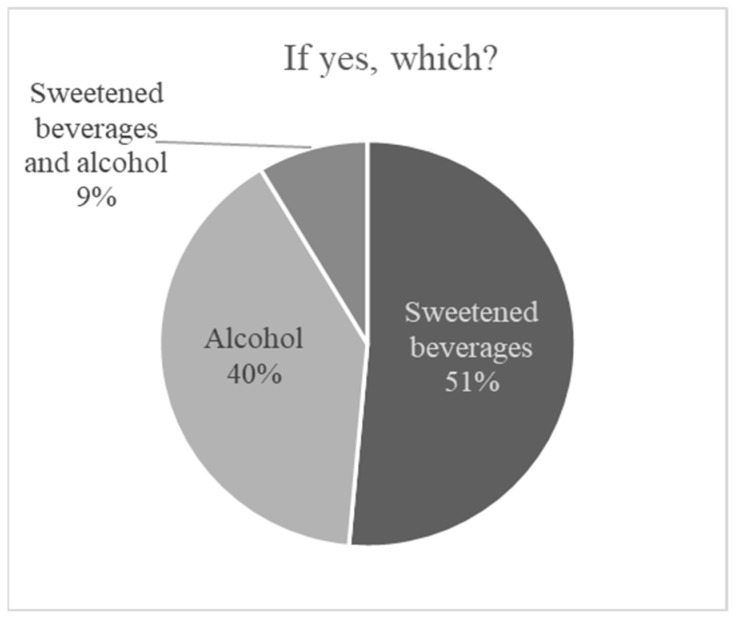
Sweetened beverages and alcohol consumption.

With respect to consumption of sugar-sweetened beverages, fruit juices, and alcohol, 64% (*n* = 63) declared that they currently did not consume any of these. The remaining 36% (*n* = 35) reported consuming at least one of these categories. Among them, 51% (*n* = 18) regularly consumed sweetened beverages or juices, 40% (*n* = 14) consumed alcohol only, and 9% (*n* = 3) consumed both sweetened and alcoholic beverages (Figure 6).

Sixty-eight percent (*n* = 67) reported regular meal consumption every 3–4 h. Lack of a fixed meal pattern was observed in 30% (*n* = 29), 2% (*n* = 2) were following an intermittent fasting 16/8 dietary model.

#### 3.5.6. Adherence to Dietary Recommendations

Sixty percent of respondents (*n* = 59) reported receiving recommendations to restrict sodium, potassium, or phosphorus intake. The remaining 40% (*n* = 39) had not received such recommendations. Among patients undergoing renal replacement therapy, four individuals reported no need to follow dietary restrictions regarding sodium, potassium, or phosphorus.

Knowledge of culinary techniques for reducing potassium and phosphorus content was declared by 43% (*n* = 42). Among them, 71% (*n* = 30) knew and applied at least two methods (soaking and double cooking). The majority (57%, *n* = 56) were not familiar with any such techniques.

**Figure 7:**
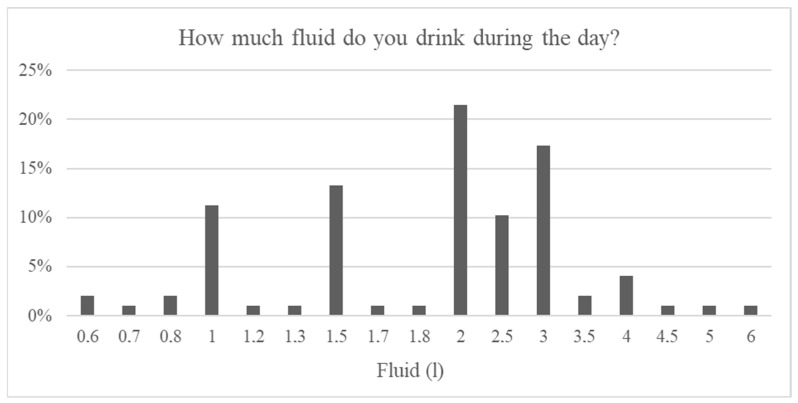
Fluid intake.

Declared recommended fluid intake values, corresponding to individual clinical status, partially differed from the actual reported intake. Daily intake of 2 L was reported by 22% (*n* = 21), including four patients with CKD stage 5D. Eighteen percent (*n* = 17) reported consuming 3 L per day, including three patients with stage 5D. Eleven percent (*n* = 10) reported 2.5 L (including one patient with stage 5D), and 13% (*n* = 13) consumed approximately 1.5 L daily. Among dialysis patients, declared daily fluid intake ranged from 0.5 to 2 L (*n* = 20). Additionally, 9% (*n* = 9) reported intake exceeding 3 L per day, including two patients with stage 5D (Figure 7).

#### 3.5.7. Multivariable Regression Analysis

In multivariable logistic regression analysis, including age, sex, and SNAQ, the model concerning knowledge about protein met statistical stability criteria (events-per-variable, EP = 24). In this model, male sex was independently associated with a lower likelihood of possessing correct knowledge about protein (β = −0.98; OR = 0.37; 95% CI: 0.14–1.00; *p* = 0.0498), indicating that men had approximately 63% lower odds of demonstrating correct knowledge compared with women. Age and SNAQ score were not significantly associated with protein knowledge.

In the model concerning knowledge about calories, age had a significant independent effect (β = −0.046; OR = 0.95; 95% CI: 0.93–0.98; *p* = 0.002). Each additional year of age was associated with approximately a 5% decrease in the odds of possessing correct caloric knowledge. Sex and SNAQ score were not significantly associated with this outcome.

## 4. Discussion

### 4.1. Nutritional Knowledge and CKD Stage

The results of this study indicate that nutritional knowledge among patients with chronic kidney disease varies considerably and is associated with disease stage. Although the majority of participants recognized the general importance of diet in CKD management, significant knowledge gaps were identified regarding specific dietary recommendations, particularly those related to phosphorus, potassium, sodium, and fluid management. These areas are clinically important because inadequate dietary control may contribute to electrolyte disturbances, metabolic complications, and further progression of kidney disease. Current recommendations from Kidney Disease: Improving Global Outcomes emphasize that nutritional education should be delivered by qualified professionals and should include individualized dietary strategies tailored to CKD stage, comorbidities, and biochemical parameters [16].

The nutritional knowledge score was significantly associated with both age and CKD stage. Higher scores were observed in patients with more advanced stages of the disease, which may reflect increased exposure to specialist care, more frequent contact with healthcare professionals, and a greater likelihood of receiving structured dietary education during disease progression.

In contrast, older age was associated with lower knowledge scores. This may be explained by factors such as reduced access to health information, lower health literacy, or age-related cognitive decline, which may affect the ability to acquire and retain complex dietary recommendations.

These findings suggest that nutritional education may be introduced later in the course of CKD, particularly when patients enter specialist nephrological care or initiate renal replacement therapy. However, this interpretation should be made with caution, as the cross-sectional design of the study does not allow for establishing causal relationships or temporal associations. Moreover, the study did not assess prior exposure to nutritional education or the timing and intensity of dietary counseling, which may have influenced the observed differences.

This pattern may suggest that nutritional education is implemented later in the disease course; however, this interpretation should be made with caution due to the cross-sectional design of the study.

Similar observations have been reported in previous studies, which indicate that patients undergoing dialysis or advanced nephrology care tend to receive more intensive dietary counseling [2]. According to current recommendations from the Kidney Disease Outcomes Quality Initiative, medical nutrition therapy should be implemented throughout all stages of CKD and not only after significant deterioration of kidney function [2]. Early education may therefore play a key role in preventing disease progression and improving long-term outcomes.

### 4.2. Knowledge Gaps in Phosphorus and Potassium Management

The analysis of specific dietary topics revealed that the greatest difficulties were related to identifying foods rich in phosphorus and potassium and understanding the principles of their restriction. These findings are consistent with previous research conducted among dialysis populations, where knowledge of phosphorus content in foods was significantly lower than knowledge of other dietary components of the renal diet [16]. Similar patterns have also been observed in non-dialysis CKD populations, suggesting that insufficient awareness of phosphorus and potassium sources represents a widespread challenge in nutritional education for kidney disease [17,18]. Considering that disturbances in phosphorus and potassium balance are associated with cardiovascular complications and increased mortality risk, improving patient knowledge in these areas should remain a priority of clinical dietary counseling.

These findings should also be interpreted in the broader clinical context of chronic kidney disease as a systemic condition associated with multiple metabolic and pathophysiological disturbances. Progressive decline in kidney function leads to significant alterations in electrolyte balance, particularly in potassium, phosphorus, calcium, and sodium homeostasis, which may contribute to cardiovascular complications and increased mortality risk. These disturbances are closely linked with other metabolic abnormalities, including inflammation, oxidative stress, and impaired energy metabolism.

Moreover, CKD frequently coexists with comorbid conditions such as hypertension, diabetes, and cardiovascular diseases, which may further accelerate disease progression and complicate clinical management. Therefore, the assessment of nutritional knowledge and dietary behaviors in CKD patients should be considered within this complex metabolic and clinical framework.

### 4.3. Discrepancies Between Knowledge and Dietary Behavior

Another important finding of this study was the discrepancy between declared nutritional knowledge and actual dietary behaviors. This phenomenon has been widely described in the literature. Several studies conducted among hemodialysis patients have demonstrated that awareness of dietary restrictions does not necessarily translate into adequate adherence to dietary recommendations, particularly regarding sodium, potassium, and fluid intake [19]. Similarly, research evaluating phosphorus education programs has shown that increased knowledge does not always lead to improved dietary phosphorus control, suggesting that additional factors—such as established eating habits, socioeconomic factors, food labeling, and the complexity of dietary restrictions—may influence patient behavior [20].

At the same time, previous studies indicate that targeted educational interventions may improve dietary adherence in CKD populations. Educational programs focusing on practical dietary strategies, including identification of phosphate additives in processed foods or techniques to reduce potassium content during food preparation, have been associated with improvements in dietary compliance and biochemical parameters [21]. These findings highlight the importance of complementing theoretical education with practical guidance that supports patients in translating knowledge into everyday dietary choices.

### 4.4. Appetite and Nutritional Status in CKD Patients

An additional aspect evaluated in this study was appetite and nutritional status. According to the SNAQ assessment, most patients reported a relatively good appetite, and only a small proportion were identified as being at risk of significant weight loss. Consistently, the SGA results indicated that the majority of participants were classified as well nourished. These findings suggest that overt malnutrition was relatively uncommon in the studied population.

However, the coexistence of adequate appetite with a high prevalence of overweight and obesity observed in this cohort highlights the complex nutritional profile of contemporary CKD populations. An interesting finding of this study is the coexistence of generally favorable nutritional status, as assessed by SGA and SNAQ, with a high prevalence of overweight and obesity in the study population. This apparent discrepancy may reflect the complex nutritional profile of patients with CKD, in whom excess body weight does not necessarily exclude the presence of qualitative nutritional deficiencies or early stages of protein–energy wasting.

Commonly used anthropometric measures, such as BMI and calf circumference, have important limitations in CKD populations. BMI does not distinguish between fat mass and lean body mass and may therefore underestimate the risk of sarcopenia or muscle wasting, particularly in patients with fluid retention. Similarly, calf circumference, although useful as a simple screening tool, may be influenced by edema and does not fully reflect muscle mass or functional status.

Therefore, the assessment of nutritional status in CKD should be based on a comprehensive approach, combining anthropometric measurements with validated clinical tools such as SGA and functional or biochemical parameters when available.

Previous studies have shown that CKD patients may present with excess body weight while simultaneously being at risk of qualitative nutritional deficiencies or early stages of protein–energy wasting [3,4,5]. Therefore, assessment of nutritional status in CKD should not rely solely on anthropometric parameters but should incorporate comprehensive tools such as SGA and validated appetite assessment instruments.

### 4.5. Use of Dietary Supplements

Another important finding is the use of dietary supplements among the participants. A substantial proportion of patients reported using supplements, particularly including vitamin D, B-complex vitamins, vitamin C, magnesium, and omega-3 fatty acids. While supplementation may be beneficial in selected clinical situations, it should be carefully individualized in CKD due to potential risks related to excessive intake of certain micronutrients or electrolyte disturbances.

For example, inappropriate supplementation may lead to hypervitaminosis, altered mineral metabolism, or interactions with prescribed medications. Current clinical guidelines emphasize that supplementation in CKD should be guided by laboratory parameters, clinical status, and medical supervision rather than self-initiated use by patients [6]. These findings highlight the need for improved patient education regarding the safe and evidence-based use of dietary supplements.

### 4.6. Limitation of the Study

This study has several limitations that should be considered when interpreting the results. First, the cross-sectional design does not allow causal relationships between nutritional knowledge and dietary behaviors to be established. Moreover, the study did not assess prior exposure to nutritional education or the timing and intensity of dietary counseling, which may have influenced the observed differences between groups. Second, the relatively small sample size may limit the statistical power of subgroup analyses. Third, the use of an author-designed questionnaire may reduce comparability with studies using standardized tools for assessing nutritional knowledge. The moderate internal consistency of the questionnaire represents a limitation, which may affect the reliability of the knowledge-related outcomes and indicates the need for further validation of the tool in future studies. Additionally, the lack of detailed data on comorbidities and broader metabolic parameters may have limited the ability to fully capture the systemic nature of CKD and its clinical complexity.

Nevertheless, the study provides valuable insight into areas with the greatest knowledge deficits among CKD patients and emphasizes the importance of early and structured nutritional education.

## 5. Conclusions

Nutritional knowledge among patients with CKD was suboptimal and varied according to disease stage, with greater deficits observed in earlier stages. The most clinically relevant knowledge gaps concerned phosphorus, potassium, sodium, and fluid management.

Knowledge levels were independently associated with demographic factors: male sex was linked to lower knowledge regarding protein intake, whereas older age was associated with poorer understanding of energy requirements.

Importantly, declared awareness of dietary recommendations did not consistently translate into appropriate dietary behaviors, indicating that knowledge alone may be insufficient to ensure adherence.

These findings highlight the need for early, structured, and stage-specific nutritional education programs tailored to patients’ clinical and demographic characteristics.

## 6. Practical Implications

1. Continuous dietary care should be considered an integral component of chronic kidney disease management at all stages of the disease rather than being limited to advanced stages. The results of this study indicate that nutritional knowledge deficits are already present in patients with early-stage CKD, highlighting the need for earlier involvement of dietitians in nephrological care.
2. Nutritional education should be delivered in a continuous and stage-specific manner, with recommendations adapted to the current stage of CKD and the treatment modality (conservative management versus renal replacement therapy). Educational strategies should include practical tools such as food lists, sample meal plans, portion guides, and culinary instructions in order to improve patient understanding and long-term adherence.
3. Particular emphasis should be placed on the practical aspects of dietary management, including culinary techniques that reduce potassium and phosphorus content in foods (e.g., soaking and double cooking). Even patients who demonstrate theoretical knowledge often fail to implement these strategies in daily dietary practice.
4. Regular assessment and monitoring of nutritional status should be incorporated into routine clinical care using anthropometric measurements and validated screening tools. This is particularly important in patients with overweight, obesity, or risk of malnutrition in order to prevent both excessive weight gain and the development of protein–energy wasting or sarcopenia. Individualized estimation of energy and protein requirements should therefore be a standard component of dietary management.
5. Effective management of electrolyte balance and dietary supplementation requires close collaboration between nephrologists and dietitians. Patients should receive clear and consistent recommendations regarding sodium, potassium, phosphorus, fluid intake, and the appropriate use of dietary supplements, which should always be individually evaluated and supervised by healthcare professionals.

## Figures and Tables

**Table 1:** Selected anthropometric parameters in the study population.

CKD Stage	1 (*n* = 9)	2 (*n* = 17)	3 (*n* = 23)	4 (*n* = 16)	5 (*n* = 22)	5D (*n* = 11)
Body weight (kg)	79.22 ± 14.90	67.21 ± 16.93	76.50 ± 16.04	83.80 ± 14.28	78.73 ± 17.13	74.53 ± 14.30
Height (cm)	171.00 ± 7.63	164.86 ± 7.72	170.55 ± 10.91	167.60 ± 11.63	171.18 ± 15.33	170.20 ± 6.10
BMI (kg/m^2^)	27.16 ± 5.37	24.61 ± 5.27	22.24 ± 10.31	29.99 ± 6.21	26.65 ± 3.32	25.76 ± 5.21
Calf circumference (cm)	40.72 ± 4.02	35.57 ± 4.22	35.14 ± 2.95	35.75 ± 4.06	35.59 ± 2.85	35.20 ± 2.68

No statistically significant differences were found in body weight, height, body mass index (BMI), or calf circumference between individual stages of chronic kidney disease. The analysis was performed using one-way analysis of variance (ANOVA). For all comparisons, *p*-values > 0.05 were obtained.

**Table 2:** Selected biochemical parameters of the study population.

CKD Stage	1 (*n* = 9)	2 (*n* = 17)	3 (*n* = 23)	4 (*n* = 16)	5 (*n* = 22)	5D (*n* = 11)	*p*-Value
GFR (mL/min/1.73 m^2^)	101.00 ± 8.00	73.43 ± 7.34	43.50 ± 8.60	20.60 ± 5.46	10.73 ± 2.00	6.47 ± 1.92	0.001
CREA (mg/dL)	0.72 ± 0.11	0.97 ± 0.14	1.59 ± 0.35	2.93 ± 0.73	4.96 ± 0.98	7.98 ± 1.72	0.001
Pi (mg/dL)	3.10 ± 0.44	3.16 ± 0.40	3.22 ± 0.57	3.79 ± 0.58	4.69 ± 0.84	5.15 ± 1.15	0.01
Ca (mg/dL)	9.29 ± 0.52	9.30 ± 0.45	9.41 ± 0.60	8.48 ± 0.48	8.79 ± 0.98	8.55 ± 1.26	0.001
K (mmol/L)	4.10 ± 0.40	4.39 ± 0.40	4.48 ± 0.51	4.78 ± 0.57	4.75 ± 0.44	5.45 ± 1.03	0.06
Na (mmol/L)	139.11 ± 1.54	138.21 ± 3.21	140.14 ± 3.00	140.00 ± 3.46	139.64 ± 2.58	139.27 ± 2.60	0.44

Differences between CKD stages were analyzed using the Kruskal–Wallis test, and when statistically significant, post hoc analysis was performed using Dunn’s test with Bonferroni correction.

## Data Availability

The original contributions presented in this study are included in the article. Further inquiries can be directed to the corresponding author.

## Abbreviations

The following abbreviations are used in this manuscript:

CKD	Chronic kidney disease
SGA	Subjective Global Assessment
SNAQ	Simplified Nutritional Appetite Questionnaire
GFR	Glomerular filtration rate
FSMP	Food for special medical purposes
IU	International unit
5DCKD	Stage 5D chronic kidney disease (patients undergoing dialysis)
BMI	Body mass index
CC	Calf circumference
VLPD	Very-low-protein diet
